# Ultrasound-Responsive Nanobubbles for Combined siRNA-Cerium Oxide Nanoparticle Delivery to Bone Cells

**DOI:** 10.3390/pharmaceutics15102393

**Published:** 2023-09-27

**Authors:** Pedram Sotoudeh Bagha, Elayaraja Kolanthai, Fei Wei, Craig J. Neal, Udit Kumar, Gillian Braun, Melanie Coathup, Sudipta Seal, Mehdi Razavi

**Affiliations:** 1 BiionixTM (Bionic Materials, Implants & Interfaces) Cluster, Department of Medicine, University of Central Florida College of Medicine, Orlando, FL 32827, USA; pedram.sotoudehbagha@ucf.edu (P.S.B.); fei.wei@ucf.edu (F.W.); melanie.coathup@ucf.edu (M.C.); 2 Advanced Materials Processing and Analysis Center, Department of Materials Science and Engineering, University of Central Florida, Orlando, FL 32826, USA; elayaraja.kolanthai@ucf.edu (E.K.); craig.neal@ucf.edu (C.J.N.); udit.kumar@ucf.edu (U.K.); sudipta.seal@ucf.edu (S.S.); 3 Department of Biological Sciences, Mount Holyoke College, South Hadley, MA 01075, USA; braun22g@mtholyoke.edu; 4 Department of Materials Science and Engineering, University of Central Florida, Orlando, FL 32816, USA

**Keywords:** ultrasound, nanobubble, Cathepsin K siRNA, cerium oxide, bone

## Abstract

This study aims to present an ultrasound-mediated nanobubble (NB)-based gene delivery system that could potentially be applied in the future to treat bone disorders such as osteoporosis. NBs are sensitive to ultrasound (US) and serve as a controlled-released carrier to deliver a mixture of Cathepsin K (CTSK) siRNA and cerium oxide nanoparticles (CeNPs). This platform aimed to reduce bone resorption via downregulating CTSK expression in osteoclasts and enhance bone formation via the antioxidant and osteogenic properties of CeNPs. CeNPs were synthesized and characterized using transmission electron microscopy and X-ray photoelectron spectroscopy. The mixture of CTSK siRNA and CeNPs was adsorbed to the surface of NBs using a sonication method. The release profiles of CTSK siRNA and CeNPs labeled with a fluorescent tag molecule were measured after low-intensity pulsed ultrasound (LIPUS) stimulation using fluorescent spectroscopy. The maximum release of CTSK siRNA and the CeNPs for 1 mg/mL of NB-(CTSK siRNA + CeNPs) was obtained at 2.5 nM and 1 µg/mL, respectively, 3 days after LIPUS stimulation. Then, Alizarin Red Staining (ARS) was applied to human bone marrow-derived mesenchymal stem cells (hMSC) and tartrate-resistant acid phosphatase (TRAP) staining was applied to human osteoclast precursors (OCP) to evaluate osteogenic promotion and osteoclastogenic inhibition effects. A higher mineralization and a lower number of osteoclasts were quantified for NB-(CTSK siRNA + CeNPs) versus control +RANKL with ARS (*p* < 0.001) and TRAP-positive staining (*p* < 0.01). This study provides a method for the delivery of gene silencing siRNA and CeNPs using a US-sensitive NB system that could potentially be used in vivo and in the treatment of bone fractures and disorders such as osteoporosis.

## 1. Introduction

Various factors, such as aging, the consumption of alcohol, and anti-inflammation medicines, can induce osteoporosis, which increases the risk of bone fractures [1]. Currently, two groups of drugs are used to treat osteoporosis: anti-resorptive drugs and pro-formatives. The former (e.g., bisphosphonates) interfere with osteoclast function, and their use can also lead to diminished in bone flexibility [2]. The latter have some limitations, such as their high cost, limited effect on the cortical bone, hormone secretion, and associated with heart disease [3,4,5]. In the bone microenvironment, osteoclast cells secret an enzyme, Cathepsin K (CTSK), which increases the degradation of the proteins of the bone, especially collagen type I [6]. Thus, one strategy to reduce bone resorption involves inhibiting this enzyme. Several CTSK inhibitors, such as Odanacatib [7], ONO-5334 [8], and MIV-711 [9], have been studied, but side effects such as heart disease and neurological diseases have limited their application [10,11].

Gene silencing using short interference RNA (siRNA) delivery can knock down bone resorption proteins [12,13]. However, achieving efficient siRNA delivery is still a significant challenge because it is a large molecule with a short lifetime and needs a carrier for delivery to the target tissue and subsequent internalization into the cells [14]. siRNA delivery has been used in bone therapies. In one study, the internalization of CTSK siRNA coated on the surface of a titanium implant improved implant angiogenesis and bone growth in vivo [15]. Nanocarriers, like bioactive glass, play an important role in siRNA internalization. In another study, a nanocarrier containing the receptor activator of nuclear factor kappa B (RANK) siRNA was delivered to silence RANK and inhibited osteoclastogenesis [16]. Furthermore, RANK siRNA delivery has been used to reduce the osteolysis caused by polyethylene particles, resulting in fewer positive cells [17]. Although local siRNA delivery is promising, systemic delivery is more favorable because osteoporosis affects the whole skeletal system [12]. In another study, an ultrasound-responsive nanobubble enhanced delivery system was used to deliver the human Na/I symporter gene into mice model skeletal muscle [18]. Also, in the same study, the gene expression was visualized using positron emission tomography (PET). Hence, a nanocarrier that can target the bone and deliver siRNA via a noninvasive method such as an ultrasound is favorable.

Ultrasound waves can penetrate different tissues based on their frequency, power, and duty cycle [19]. Moreover, ultrasound-based techniques are beneficial for bone formation and fracture healing, making them ideal for bone disease treatment [20,21]. Several ultrasound-responsive nanocarriers, including liposomes and micelles, have been investigated for releasing therapeutics [22,23,24,25]. Ultrasound waves can stimulate carriers by expanding them or by applying mechanical force to aid in the release of their contents [26]. Ultrasound-responsive nanocarriers have been used for siRNA delivery. Guo et al. [27] increased siRNA delivery to brain tumors by 10 times by designing a lipid–polymer hybrid nanoparticle combined with microbubbles. In addition to cellular uptake, lysosomal trapping is an obstacle to siRNA delivery efficiency. A hybrid nano assembly platform irradiated with ultrasound-generated singlet oxygen has been shown to help siRNA to escape from lysosome, leading to gene silencing [28]. So, a promising delivery method could be realized through the use of an ultrasound-mediated nanobubble delivery system that can encapsulate siRNA and release it in a controlled manner via ultrasound stimulation. Nanobubble gene delivery has been used for treating lung cancer [29] and melanoma [30], but it can also be used for treating osteoporosis by encapsulating CTSK siRNA [31]. Another trigger of osteoporosis due to the low estrogen during menopause is oxidizing enzymes such as NOX1 via reactive oxygen species (ROS) [32]. ROS is an essential factor for bone resorption in a healthy condition; however, under conditions like menopause, it can induce cell apoptosis in osteoblasts and osteocytes and increase osteoclast formation [33]. Ceria (CeO_2_) has both antioxidant and pro-oxidant properties due to its dual oxidation states (Ce^3+^ and Ce^4+^), which enable it to act as a superoxide oxidase and catalase [34]. In one study, these features made nanoceria a suitable candidate for osteoporosis treatment by suppressing ROS [35]. Interestingly, these materials may be synthesized so that their pro- and antioxidant surface chemistries are disproportionate. This surface chemistry tuning has been demonstrated numerous times despite the fact that the antioxidant properties outweigh the pro-oxidant properties when administered both in vitro and in vivo [36,37]. In comparison to natural antioxidant species, cerium oxide nanoparticles (CeNPs) present the advantages of catalytic activity (e.g., relative to endogenous molecular species such as glutathione) and significant physicochemical stability (i.e., relative to enzymes like superoxide dismutase). In particular, the stability of CeNPs is crucial in biomedical applications, involving focused irradiation, such as via ultrasound, where local heating can lead to the hydrolysis of less stable organic species. The physiochemical and biological properties of CeNPs can be tailored via synthesizing methods such as precipitation, hydrothermal synthesis, green synthesis, spray coating, sol–gel, etc. [38]. Aging CeNPs with peroxide is a method that has been used to stabilize CeNPs against the effect of pH and temperature change [39]. In a study on bone disease, CeNPs coated with dextran were used to treat osteosarcoma, and they protect normal cells from free radicals [40]. Also, there is evidence to suggest that, in the presence of CeNPs, T-cells can release more interleukin-2 (IL-2) and tumor necrosis factor-α (TNF-α), helping to provide more effective cancer therapy [41]. Reducing reactive oxygen species (ROS) protects osteoblasts against oxidative damage generated from osteoclasts, so it is beneficial in osteoporosis treatment [35,42]. In addition to CeNPs particles, CeNPs bioglass and CeNPs hydroxyapatite scaffolds have been shown to improve osteogenic activity by affecting the Smad-dependent-bone morphogenic protein signaling pathway and the upregulation of bone markers such as osteocalcin (OCN), alkaline phosphatase (ALP), and type I collagen (COL-1) [43,44]. Although most drugs for osteoporosis treatment do not initiate bone formation, CeNPs can have both protective effects on osteoclastogenesis and induce new bone formation [45]. For instance, in one study, ovariectomy (OVX) rats implanted with Ce-HA in their tibia showed higher bone formation after 30 days (analyzed via Raman and X-ray microtomography) [46]. Another merit of CeNPs is their angiogenic ability, which helps the formation of new vasculatures for supplying nutrients, facilitating enhanced cell migration and better cell growth [47,48,49].

Because the administration of CeNPs is mostly carried out through vein injection, it can diffuse into the capillaries of endothelial cells with 6 nm pore size and lymph. Also, CeNPs less than 6 nm can be excreted by the urinary system in vivo [50] or they can accumulate in the liver and spleen [51]. Although CeNP injection did not damage tissues in mice after 100 days in one specific study [52], it is crucial to target CeNPs to the bone, and NB is a platform that can be used as a nanocarrier. Moreover, the encapsulation of CeNPs in NB and surface functionalization with PEG can be promising for escaping from the immune system [53]. Although most of the studies in the literature have focused on inactivating osteoclasts to treat osteoporosis, the aim of this study was to develop an ultrasound-responsive nanobubble that can deliver CTSK siRNA and CeNPs to suppress osteoclast differentiation and promote osteogenesis in vitro synergistically. In our study, a mixture of CTSK siRNA and CeNPs was loaded onto NB and characterized using transmission electron microscopy (TEM) and X-ray photoelectron spectroscopy (XPS). Next, the mineralization test, Alizarin Red Staining (ARS), was performed on differentiated human bone marrow-derived mesenchymal stem cells (BMSC). Finally, osteoclastogenesis was evaluated via the tartrate-resistant acid phosphatase (TRAP) staining of human osteoclast precursors (OCP).

## 2. Materials and Methods

### 2.1. Materials

Cerium nitrate hexahydrate (purity 99.999; Ce(NO_3_)_3_.6H_2_O), hydrogen peroxide, Paraformaldehyde (PFA), and fluorescein isothiocyanate (FITC) were purchased from Thermo Fisher Scientific (Waltham, MA, USA). Albumin from human serum, Cetylpyridinium chloride, sodium phosphate, β-glycerophosphate, L-ascorbic acid 2-phosphate, dexamethasone, and Alizarin Red S solution were purchased from Millipore Sigma (Burlington, MA, USA). Ammonium hydroxide (NH_4_OH) was purchased from Alfa Aesar (Haverhill, MA, USA). Sodium periodate, Epichlorohydin, 30% ammonium hydroxide, and sodium hydroxide (NaOH) were purchased from Sigma-Aldrich. Human osteoclast precursors (2T-110), OCP^MT^ basal media, and L-glutamine were purchased from Lonza. Penicillin (10,000 units/mL), streptomycin (10,000 µg/mL), and human trance recombinant protein (RANKL) were purchased from Gibco (Billings, MT, USA). Perfluorohexane (C_6_F_14_) was purchased from Fluoromed (Round Rock, TX, USA). Cathepsin K siRNA (CTSK siRNA) and Cathepsin K siRNA labeled with fluorescein isothiocyanate (CTSK siRNA-FITC) were purchased from integrated DNA technology (IDT) (Coralville, IA, USA). Control siRNA (SC-37007) and control siRNA labeled with Cy3 (siRNA-Cy3) were purchased from Santa Cruz Biotechnology, Inc. (Dallas, TX, USA), and Cell Signaling Technology (Danvers, MA, USA), respectively. Human bone marrow-derived mesenchymal stem cells (hMSCs, PCS-500-012) were purchased from ATCC (Manassas, VA, USA). Thiazolyl Blue Tetrazolium Bromide (MTT 98%) was purchased from Acros Organics (Morris Plains, NJ, USA). The transfection reagent we used (HiPerFect) was purchased from Qiagen Science (Germantown, MD, USA). Recombinant human M-CSF protein was purchased from Novous Biologicals (Centennial, CO, USA). TRAP staining kit was purchased from CosmoBio (Tokyo, Japan). Dimethylsulfoxide (DMSO) was purchased from Fisher BioReagents (Pittsburgh, PA, USA). DMEM Low Glucose (Dulbecco’s Low Glucose Modified Eagle’s Medium) was purchased from Cytiva (Marlborough, MA, USA). Fetal bovine serum (FBS) was purchased from Neuromics (Minneapolis, MN, USA). Neutral buffered formalin (NBF) was purchased from Epredia (Kalamazoo, MI, USA). The fluorescent dye used for DNA staining (Hoechst 33342) was purchased from Bio-Techne Corporation (Minneapolis, MN, USA).

### 2.2. Preparation of Cerium Oxide Colloidal Solution and Fluorescent Labeling

Preparation of cerium oxide: Cerium oxide nanoparticles were synthesized according to our previous procedures (published elsewhere) [54]. A total of 5 mM Ce(NO_3_)_3_·6H_2_O was dissolved in 50 mL of deionized water using a magnetic stirrer. Then, 2 mL of 3% hydrogen peroxide was added to Ce(NO_3_)_3_.6H_2_O solution for oxidation to cerium oxide. When hydrogen peroxide was added, the solution changed from colorless to yellow, indicating oxidation to cerium oxide. The color of the solution gradually decreased with increasing aging time at room temperature. After 7 to 8 weeks of aging, this solution became completely colorless, indicating the formation of cerium oxide nanoparticles and that it was ready for use in experiments.

CeNPs-NH_2_ preparation for FITC labeling: A total of 2.48 g of Ce(NO_3_)_3_.6H_2_O was dissolved in 100 mL of deionized water under magnetic stirring. Then, 10 mL of 1 N NH_4_OH was added dropwise under stirring at 450 rpm on a magnetic stirring. The solution was stirred for 4 h. Subsequently, the resulting particles were collected and purified through centrifugal washing (3×) with deionized water. The final product was re-suspended in deionized water and ultra-sonicated to facilitate particle dispersion. The final concentration of the particles was determined via multiple measurements of the particle dry mass from sample aliquots. Amine surface modification was achieved using an epichlorohydrin-mediated process.

A total of 250 mg of CeNPs, synthesized per the procedure above and dried overnight at 60 °C, were added to a 10 mL solution of 0.1 M NaOH and stirred for 5 min. Then, 5 mL of distilled epichlorohydrin was added to this solution, followed by 0.5 mL of 2 M NaOH and stirring for 6 h. The particles were then collected and washed (3×) with deionized water using centrifugation. The isolated pellet was then re-suspended in 25 mL of 30% NH_4_OH and stirred for several minutes. Finally, the particles were washed (3×) with deionized water via centrifugation and re-suspended in deionized water.

CeNPs-FITC labeling: CeNPs-FITC were synthesized using CeNPs-NH_2_ synthesized via a precipitation reaction (carried out according to the above procedures) and a FITC surface labeling protocol. Briefly, 1 mL aqueous solution of sodium periodate (initial concentration: 10 mg/mL) was added to 10 mL aqueous suspension of 5 mM (initial concentration) CeNPs-NH_2_. Next, the solution was stirred at 400 rpm for 18 h and subsequently dialyzed over 3 days (exchange of dialysate every 12 h). A total of 1 mL of 10 mM FITC (prepared in DMSO) was then added to the dialyzed particle suspension and allowed to mix for several minutes. A total of 5 mL of a pH 8.5 bicarbonate buffer was subsequently added, and the solution was placed on a rocker table for 6 h. The final solution was purified by first washing the particles with water via centrifugation in triplicate and subsequently via dialysis for three additional days (dialysate exchanged every 12 h).

### 2.3. Nanobubble Preparation and Characterization

Nanobubbles loaded with cathepsin K siRNA (CTSK siRNA) and CeNPs (NB-(CTSK siRNA + CeNPs)) were synthesized via the addition of 40 mg Albumin from human serum, 300 µL perfluorohexane (C_6_F_14_), 43 µL of 1000 nM CSTK siRNA, and 4 mL of 860 µg/mL CeNPs. For cell internalization, 25 µL of siRNA-Cy3 (10 µM) was added to 40 mg Albumin from human serum and 300 µL perfluorohexane (C_6_F_14_) to prepare NB-250 nM siRNA-Cy3. The mixture was sonicated in an ice bath using an ultrasonic probe (Fisherbrand™ Model 120 Sonic Dismembrator) with the following parameters: frequency = 20 kHz, power = 120 W, duration = 2 min and 30 s (5 cycles of 30 s ON and 15 s OFF). Then, the solution was centrifuged at 15,000 rpm (Optima XPN-100 Ultracentrifuge, Beckman Coulter (Brea, CA, USA) at 22.5 °C and washed with PBS once [31]. Previously, sonication was used to load siRNA into extracellular vesicles, and the optimum time to avoid degradation was 30 s [55]. Thus, this was also selected as our sonication time.

After synthesizing NB-(siRNA labeled with FITC) and NB-(CeNPs labeled with FITC), the supernatant was collected separately to calculate the NBs surface adsorption efficiency (AE%), i.e., the amount of siRNA or CeNPs adsorbed on the surface of nanobubbles/amount of siRNA or CeNPs added × 100, and the loading efficiency (LE), i.e., weight of siRNA or CeNPs loaded in nanobubbles ((µg)/weight of final nanobubble pellet (mg)). The amounts of FITC-labeled siRNA and CeNPs were measured according to their fluorescent intensity via fluorescence spectroscopy (SpectraMax iD5, Molecular Devices, San Jose, CA, USA) at excitation and emission wavelength of 485 and 535 nm. The intensity was extrapolated with a standard calibration curve using a linear regression method. To illustrate the loading of CeNPs in the nanobubble’s shell, 1 mg/mL of NB loaded with CeNPs labeled with FITC (0, 8, 80, and 320 nM) was stimulated via another probe (Win Health Medical Ltd., Jedburgh, UK) via a low-intensity pulsed ultrasound (LIPUS, pulsed = 50%, duration = 4 min, frequency = 1 MHz, and power = 1 W/cm^2^) to make it detectable using a fluorescent microscope. Then, it was observed via fluorescence microscopy (BZ-X800, Keyence, Osaka, Japan) after 2 h of incubation at room temperature after LIPUS. The release profile of CTSK siRNA and CeNPs (both labeled with FITC) after LIPUS stimulation with the same aforementioned parameters were measured for 1 mg/mL of NB without changing the media after 1, 3, and 7 days at 37 °C. The supernatant was collected and centrifuged at 4000 rpm for 5 min, and the fluorescent intensity of the supernatant was measured (excitation = 485 nm; emission = 535 nm).

### 2.4. Nanomaterial Characterization

Using a 300 kV Philips Tecnai high-resolution transmission electron microscope (HR-TEM), the size and morphology of bare CeNPs and mixed CTSK siRNA/CeNPs were analyzed. The diluted nanoparticles were coated on holey carbon TEM grids and dried at room temperature before imaging. Image analyses were carried out using DigitalMicrograph software 3.6.5 (GATAN, Pleasanton, CA, USA). UV-Vis spectrophotometry (Lambda 650, PerkinElmer, Waltham, MA, USA) was examined for pure and mixed CTSK siRNA/CeNPs to observe the change in Ce (III) and Ce (IV) states upon the addition of siRNA molecules. The UV-Vis spectrum was recorded between 220 nm and 400 nm. The cerium oxide surface states and chemical compositions were analyzed using X-ray photoelectron spectroscopy (XPS). An ESCALAB-250Xi spectrometer was used to record the XPS spectrum for these nanoparticles. During the measurement, a spectrometer was used to generate the X-ray from a monochromatic Al-Kα radiation source below 7 × 10^−9^ mbar vacuum condition operating at a power of 300 W (15 kV, 20 mA). The X-ray beam’s spot size was fixed at 600 μm for all the samples, and the C1s peak at 284.6 eV was used as a reference for binding energy calibration. The XPS spectra were analyzed using Thermo Avantage V5.9904 software (Thermo Scientific, Waltham, MA, USA). Pure CeNPs, CTSK siRNA, and mixed CTSK siRNA/CeNPs samples were coated on a gold substrate and dried overnight at room temperature.

The antioxidant properties of CTSK siRNA (50 nM), a mixture of CTSK siRNA/CeNPs (50 nM/5 µg/mL and 50 nM/10 µg/mL), and various concentrations of pure CeNPs (5, 10, and 20 µg/mL) were examined using the superoxide dismutase (SOD) mimetic assay kit. Briefly, 20 µL of pure CeNPs, CTSK siRNA, and mixed CTSK siRNA/CeNPs were added to a 96-well plate, followed by 200 µL of water-soluble tetrazolium solution that had been mixed well using a pipette. In accordance with the manufacturer’s instructions. we maintained three blank samples. A total of 20 µL of deionized water was added to the blank 1 and blank 3 wells. Furthermore, 20 µL of dilution buffer was added to the blank 2 and blank 3 wells. Finally, 20 µL of enzyme solution was added to each sample condition and the blank 1 well. After adding the enzyme solution, reaction kinetics-related absorbance values were recorded at 450 nm using a plate reader. Measurements were performed for each condition sample, and the derived averages were used to plot a time versus absorbance graph.

### 2.5. Cell Toxicity

Human bone marrow-derived mesenchymal stem cells (hMSCs) were cultured for 1 day in a 96-well plate (10,000 cells per well). hMSCs were then cultured alone as control (0 mg/mL) or with NB at different concentrations (0.25, 0.5, 1, and 5 mg/mL) for 1 day. The metabolic activity of the hMSCs was assessed using Thiazolyl Blue Tetrazolium Bromide (MTT). Additionally, 20 µL of MTT was added to each well and incubated for 4 h. Next, the dissolving of the formazan crystals was performed via the addition of 50 µL of DMSO. The absorbance was finally read by using a microplate reader (SpectraMax iD5, Molecular Devices, San Jose, CA, USA) at 570 nm.

### 2.6. In Vitro Mineralization

The mineralization of human bone marrow-derived mesenchymal stem cells (hMSCs) was determined using Alizarin Red Staining (ARS) after 28 days of culture. First, hMSCs were cultured in a 24-well plate at a density of 100,000 cell/well in osteogenic media prepared using DMEM with 10% Fetal bovine serum (FBS), 2 mM β-glycerophosphate, 100 μM L-ascorbic acid 2-phosphate, and 10 nM dexamethasone. The cells were incubated for 28 days and treated every 3 days with the following groups: (i) 2.5 nM CTSK siRNA, (ii) 2.5 nM control siRNA, (iii) 1 µg/mL CeNPs, (iv) mixture of 1 µg/mL CeNPs and 2.5 nM CTSK siRNA, (v) 1 mg/mL of NB loaded with a mixture of 1 µg/mL CeNPs and 2.5 nM CTSK siRNA (NB-(CTSK siRNA + CeNPs)), and (vi) control negative (control (hMSCs)). Before being added to the cells, group (v) was stimulated via LIPUS, and all groups except CeNPs were treated with 1 vol.% transfection reagent for 5 min in a serum-free basal media.

After 28 days, the cells were fixed with 4% Paraformaldehyde (PFA) solution for 20 min at room temperature. After washing the cells with deionized water, 2% Alizarin Red S solution with 4.1 pH was added to each well to cover the cells, which were then incubated at room temperature for 20 min. After washing the cells thoroughly, images were captured using an optical microscope (BZ-X800, Keyence, Osaka, Japan). Then, 10% Cetylpyridinium chloride in 10 mL of sodium phosphate 10 mM pH 7 was added to dissolve the red crystals. After 15 min of incubation at room temperature, 100 µL of the solution was transferred to a 96-well plate and quantified by measuring the optical density at 562 nm.

### 2.7. Cell Internalization

Human osteoclast precursors were cultured in a 96-well plate with 3000 cells/mL in OCP^MT^ basal media containing 10% fetal bovine serum (FBS), 1% L-glutamine (200 mM), 1% penicillin and streptomycin (10,000 units/mL penicillin and 10,000 µg/mL streptomycin), 33 ng/mL of recombinant human M-CSF protein (0.25 mg/mL), and 66 ng/mL of human trance) recombinant protein (10 µg, RANKL). After 7 days of incubation in 5% CO_2_ at 37 °C, media were removed, and the cells were incubated for 24 h with 100 µL of nanobubbles loaded with 250 nM Cy-3 labeled siRNA (NB-250 nM siRNA-Cy3) after LIPUS simulation with 1 vol.% transfection reagent and post incubated for 72 h at 37 °C. Before imaging, the media were replaced with Hoechst stock solution (10 mg/mL) diluted in PBS (1:2000) and incubated for 10 min in dark. Then, the solution was removed and washed 3 times with PBS and imaged via the use of a fluorescent microscope (BZ-X800, Keyence, Osaka, Japan).

### 2.8. Osteoclastogenesis

Human osteoclast precursors were cultured in a 24-well plate with 100,000 cells/mL in OCP^MT^ basal media containing 10% fetal bovine serum (FBS), 1% L-glutamine (200 mM), 1% penicillin and streptomycin (10,000 units/mL penicillin and 10,000 µg/mL streptomycin), 33 ng/mL of recombinant human M-CSF protein (0.25 mg/mL), and 66 ng/mL of human trance) recombinant protein (10 µg, RANKL). After 1 day of incubation in 5% CO_2_ at 37 °C, the cells were treated with the same experimental groups explained for the in vitro mineralization every 3 days. To assess osteoclast formation, tartrate-resistant acid phosphatase (TRAP) staining was performed after 7 days via the use of the TRAP staining kit. First, the media were removed, and each well was washed with 500 µL of PBS. Osteoclasts were then fixed for 5 min with 250 µL of 10% Neutral buffered formalin (NBF) and washed 3 times with 1 mL of deionized water. Next, 250 µL of Chromogenic substrate was added to the wells and incubated for 1 h at 37 °C. Finally, the cells were washed with deionized water and imaged using a microscope (BZ-X800, Keyence, Osaka, Japan). Quantification was performed using image analyzer software (version 153k, ImageJ, Bethesda, MD, USA), and the results were reported as TRAP-positive cell number per mm^2^ and TRAP-positive cell size (µm^2^/cell). To calculate cell size, three region of interests (1 mm^2^) were selected and the surface area of the cells were calculated. Control −RANKL and control +RANKL served as the negative and positive control groups, respectively.

### 2.9. Statistical Analysis

SOD analysis was performed in triplicate (n = 3) for each sample condition, while XPS and TEM data analyses were performed from single spectra and images (n = 1) for each sample condition. The release tests were performed with 8 replicates (n = 8) for CTSK siRNA and 6 replicates (n = 6) for CeNPs. A cytotoxicity assay and ARS were performed in triplicate (n = 3). For the TRAP staining measurements, the cells were counted in 3 (1 mm^2^) regions of interest across 5 replicates (n = 5) for cell counting and 3 replicates (n = 3) for cell surface area. The results are expressed as mean ± standard deviation (SD). A one-way ANOVA (analysis of variance) with Tukey’s post hoc test was used for statistical analysis (GraphPad Prism version 9.4.0, San Diego, CA, USA). Data were considered statistically significant at *p* < 0.05.

## 3. Results

**Figure 1:**
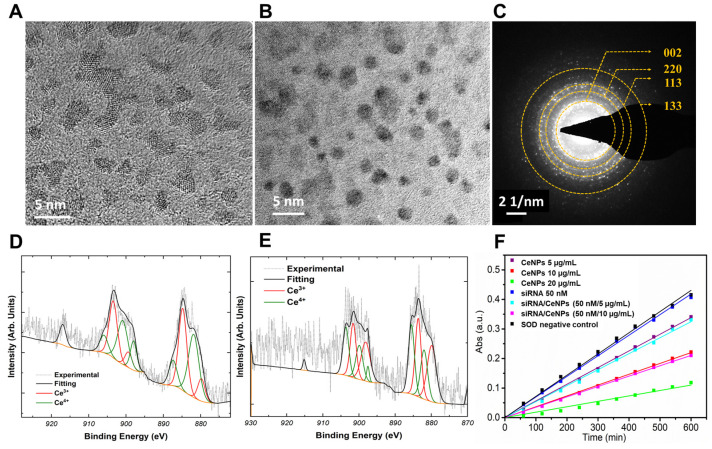
(**A**,**B**) show HR-TEM images of CeNPs and mixed CTSK siRNA/CeNPs. We observed that the cerium oxide particles possessed an average diameter of 3–5 nm (**A**). The particles appear spherical in shape, with uniform dispersity after CTSK siRNA conjugation with CeNPs (**B**). There was no significant change in particle size following the mixing. (**C**) shows a corresponding SAED ring pattern that confirms the presence of fluorite crystal-structured CeNPs. The rings from the pattern were determined to specifically correspond with the (002), (220), (113), and (133) planes. X-ray photoelectron spectroscopy (XPS) of control CeNPs (**D**) and a mixture of CTSK siRNA/CeNPs samples (**E**). (**F**) shows the superoxide dismutase (SOD) activity of different concentrations of CeNPs and a mixture of CTSK siRNA/CeNPs samples. There was no SOD activity for the CTSK siRNA molecules, as can be observed in this graph. The graph shows the positive correlation between particle concentrations and measured SOD activity. Furthermore, adding CTSK siRNA to CeNPs did not significantly affect the SOD activity. SOD negative control is a zero SOD activity reference control provided by the manufacturer (Dojindo laboratories, Kumamoto, Japan).

The particle size and morphology of synthesized CeNPs and CTSK siRNA/CeNPs mixture were analyzed using TEM techniques. A particle size of approximately 3 to 5 nm with uniform dispersion was observed for the control CeNP samples (Figure 1A). In contrast, the CTSK siRNA/CeNPs mixture samples showed spherical particles with a pronounced dark contrast in the TEM images. This dark contrast in the TEM images is ascribed to the density of organic CTSK siRNA conjugated to the CeNPs surface [56]. Despite this, individual particles are still observable in the micrograph (Figure 1B); the size of the particles was around 3–6 nm (estimated using ImageJ), similar to pure CeNPs. The corresponding selected area electron diffraction (SAED) pattern for the CTSK siRNA/CeNPs sample, shown in Figure 1C, possesses a ring pattern corresponding to (002), (220), (113), and (133) planes specific to the fluorite structure of cerium oxide. Chemical state analysis was performed for both the control CeNPs and the CTSK siRNA/CeNPs samples according to cerium redox state composition. X-ray photoelectron spectroscopy (XPS) measurements and analysis in the binding energy region characteristic of the Ce3d orbitals are presented in Figure 1D,E. The measurement signal was de-convoluted into multiple peak contributions, with spin–orbit coupling producing Ce3d_5/2_ and Ce3d_3/2_ envelopes [57,58]. Fitted peaks are ascribed to varied electronic configurations related to the Ce^3+^ and Ce^4+^ states. The fraction of cerium sites measured in reduced form was determined to be ~60% (or Ce^3+^/Ce^4+^: 1.5) for both the CeNPs and the CTSK siRNA/CeNPs samples (calculated as the sum of integrated Ce^3+^ peak signal intensities and normalized to the total sum over all cerium-related peaks). It should be noted that other studies have observed that X-ray irradiation power and vacuum exposure time can influence the degree of cerium reduction. Therefore, we limited the exposure of the test samples to these conditions. Additionally, the measured values are comparable to previous measurements for similar formulations. Interestingly, X-ray absorption studies in the existing literature suggest a near-absence of reduced site cerium in colloidal CeNPs [59,60]. These studies contend that redox occurs at the particle surface through electron occupation in configurations of mixed Ce-O orbital characters [59]. Herein, we consider only the measured value of Ce^3+^/Ce^4+^ as a qualitative metric of surface chemistry, given the substantial published literature relating this value with enzyme-mimetic character [61].

The pure CeNPs, CTSK siRNA, and CTSK siRNA/CeNPs solution were evaluated for SOD activity using a Dojindo assay kit [57,58]. A xanthine oxidase/xanthine reaction system was used to estimate the superoxide anion scavenging potential of these samples. Figure 1F shows the time vs. absorbance of xanthine reaction towards reactive oxygen species (ROS). Three different concentrations of control CeNPs (5, 10, and 20 µg/mL) and two different concentrations of the mixture of CTSK siRNA/CeNPs (50 nM/5 µg/mL and 50 nM/10 µg/mL) were used for this assay. The slope values for each sample were determined from the data presented in Figure 1F and then used to calculate the sample SOD activities according to the following: SOD activity = ((slope of absorbance change of SOD blank − slope of absorbance change of samples)/(slope of absorbance change of SOD blank)) × 100 % [57]. The percentages of SOD activity were 15 ± 2, 42 ± 1, and 71 ± 1% for 5, 10, and 20 µg/mL, respectively. Furthermore, the percentages of SOD activity for CTSK siRNA mixed with 5 and 10 µg/mL of the CeNP samples were 16 ± 2 and 73 ± 2%.

**Figure 2:**
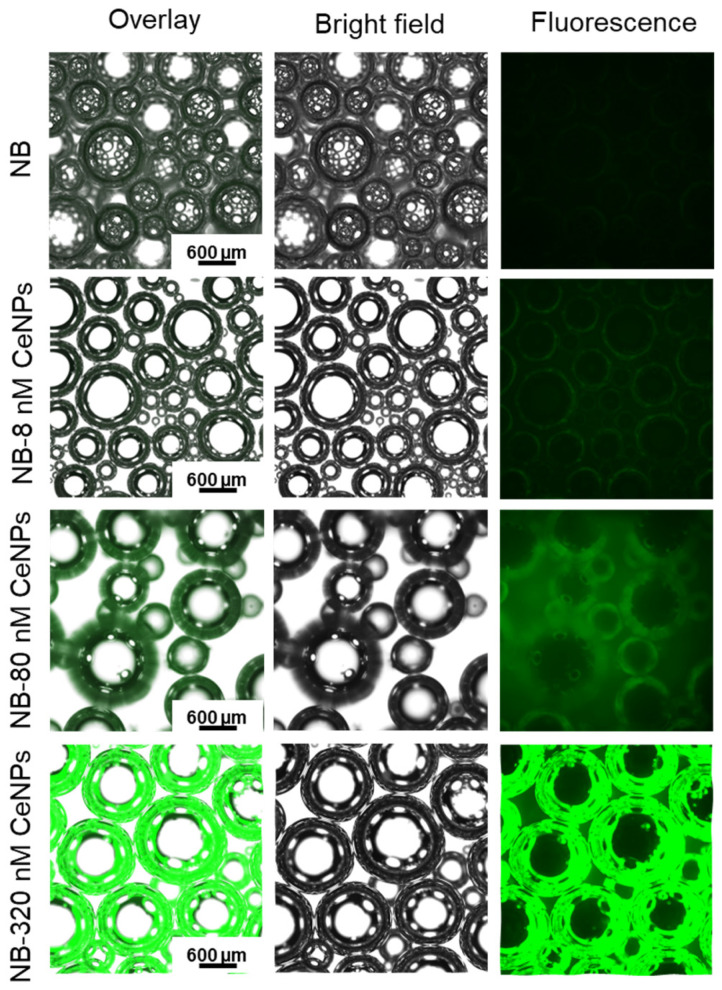
Fluorescent and bright field microscopy of nanobubbles loaded with 0, 8, 80, and 320 nM of FITC-labeled CeNPs after 2 h of LIPUS stimulation for 4 min (1 MHz frequency; 1 W/cm^2^).

**Figure 3:**
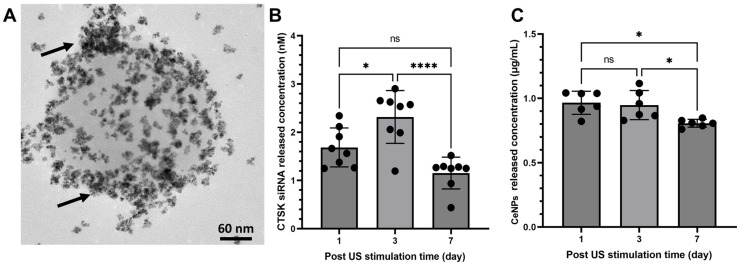
(**A**) Transmission electron microscopy of nanobubbles loaded with CeNPs (black arrows show CeNPs adsorbed on a nanobubble) and (**B**,**C**) release profile of siRNA and CeNPs from 1 mg/mL of US-stimulated NB after 1, 3, and 7 days of incubation at 37 °C. Significant differences were determined using a one-way ANOVA and Tukey’s post hoc test. ns = not significant (*p* > 0.05). *: *p* < 0.05; ****: *p* < 0.0001.

To confirm CeNPs incorporation within NBs, fluorescent molecule-modified CeNP-filled NBs were prepared and viewed using fluorescence microscopy. Figure 2 demonstrates the loading of NB with the CeNPs-FITC that was stimulated using the LIPUS for 4 min (1 MHz frequency; 1 W/cm^2^), and the images were captured after 2 h of bubble expansion after LIPUS using a fluorescent microscope. The green, fluorescent color was stronger in the bubble’s shell when 320 nM of CeNPs was loaded into the NB compared to 0, 8, and 80 nM CeNPs, indicating the adsorption of CeNPs on the surface of the NB. Also, a TEM image confirmed the presence and distribution of the CeNPs in the NB (Figure 3A). The size of the nanobubbles before US stimulation was determined to be 150 ± 50 nm via TEM; however, it showed a high polydispersity [31]. AE% was calculated based on the fluorescent intensity of the supernatant after NB synthesis, and it was 25.44 ± 5.38% for CTSK siRNA and 17.28 ± 1.94% for the CeNPs. Also, LE was calculated to be 2.57 ± 0.54 µg/mg and 1.86 ± 0.21 µg/mg for CTSK siRNA and the CeNPs, respectively. To quantify the amount of CTSK siRNA and CeNPs released from the nanobubbles, the fluorescent intensity was measured for 1 mg/mL of the following two groups: NB-(CTSK siRNA-FITC) and NB-(CeNPs-FITC) (Figure 3B). The amount of released CTSK siRNA increased from 1.69 ± 0.4 nM (1 day) to 2.32 ± 0.55 nM (3 days) and then decreased to 1.15 ± 0.33 nM (day 7). On the other hand, the maximum release of CeNPs was observed to be 0.97 ± 0.09 µg/mL on the first day of stimulation but decreased to 0.95 ± 0.11 µg/mL and 0.81 ± 0.03 µg/mL after 3 and 7 days, respectively (Figure 3B).

**Figure 4:**
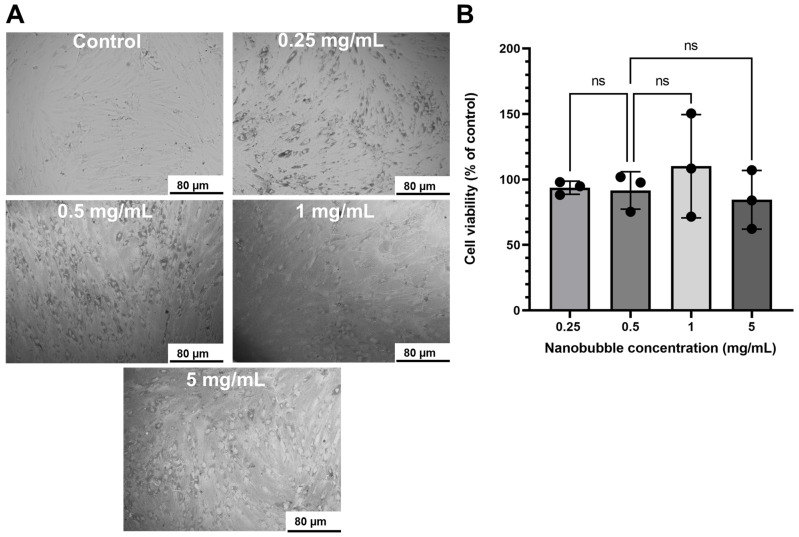
NB Biocompatibility. (**A**) Bright-field microscopy images of hMSCs cultured with different concentrations of NB (0.25 mg/mL, 0.5 mg/mL, 1 mg/mL, and 5 mg/mL); (**B**) the relevant cell viability of hMSCs cultured with NB for 1 day. Significant differences were determine using a one-way ANOVA and Tukey’s post hoc test. ns = not significant (*p* > 0.05).

The biocompatibility of the nanobubbles with the hMSCs was analyzed by culturing the cells with different NB concentrations (0, 0.25, 0.5, 1, and 5 mg/mL; Figure 4A). To evaluate the metabolic activity of the hMSCs, an MTT assay was performed after 1 day of incubation. The cell viability did not change significantly (*p* > 0.05) by increasing the concentration of NB from 93.7 ± 5% for 0.25 mg/mL to 84.5 ± 22.4% for 5 mg/mL (Figure 4B). However, by increasing the concentrations to 5 mg/mL, there was a deposition of NB residues that could result from the degradation of PFC and albumin; thus, 1 mg/mL NB was selected for further analysis. Also, the maximum amounts of CTSK siRNA and CeNPs that showed releases from 1 mg/mL of NB were 2.32 ± 0.55 nM and 0.97 ± 0.09 µg/mL. Therefore, round-up values of 2.5 nM CTSK siRNA and 1 µg/mL CeNPs were chosen for further in vitro studies.

**Figure 5:**
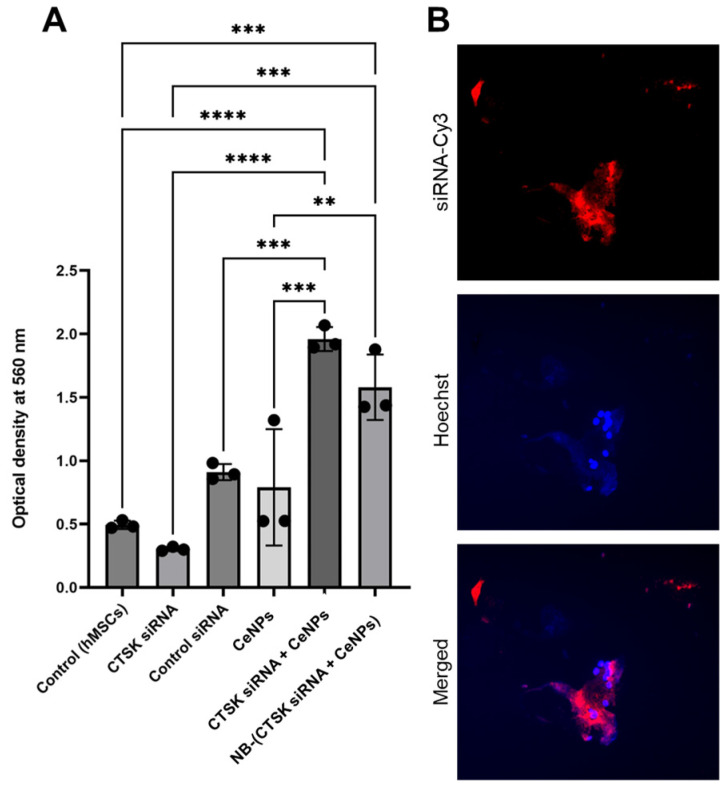
(**A**) Quantification of mineralization/calcium deposition via absorbance measurements of solubilized Alizarin Red S from the Alizarin Red Staining (ARS) of hMSCs cultured with the samples for 28 days: control negative (control (hMSCs)), 2.5 nM CTSK siRNA, 2.5 nM control siRNA, 1 µg/mL CeNPs, 1 µg/mL CeNPs + 2.5 nM CTSK siRNA, NB-(2.5 nM CTSK siRNA + 1 µg/mL CeNPs). Significant differences were determined using a one-way ANOVA and Tukey’s post hoc test. **: *p* < 0.01, ***: *p* < 0.001, ****: *p* < 0.0001. (**B**) Fluorescence microscopy image of NB-250 nM siRNA-Cy3 in osteoclast cells (magnification = 10×). NB-250 nM siRNA-Cy3 in red; Hoechst-stained osteoclast nuclei in blue. Cells were transfected for 24 h with 1 vol.% transfection reagent (HiPerFect) and subsequently incubated for 72 h at 37 °C.

ARS staining showed that the incubation of hMSCs with 1 µg/mL CeNPs + 2.5 nM CTSK siRNA and NB loaded with 2.5 nM CTSK siRNA + 1 µg/mL CeNPs results in a higher calcium deposition and significantly higher OD values (1.96 ± 0.09 and 1.58 ± 0.26, respectively) compared to the control (0.49 ± 0.03) (*p* < 0.0001 and *p* < 0.001, respectively; Figure 5A). The mineralization efficiency of the mixture of CTSK siRNA and CeNPs is higher than the CTSK siRNA or CeNPs alone (1.96 ± 0.09 vs. 0.3 ± 0.16 and 0.79 ± 0.46, respectively, *p* < 0.001; Figure 5A). CeNP internalization may facilitate the process of siRNA internalization due to endocytosis. It is also possible that siRNA was conjugated to CeNPs in a mixture. This would have enhanced the efficiency of siRNA transfection. While NB-(CTSK siRNA + CeNPs) showed a lower OD value compared to the mixture of CTSK siRNA and CeNPs (1.58 ± 0.26 vs. 1.96 ± 0.09), this was not statistically significant (*p* > 0.05).

The internalization of NB-250 nM siRNA-Cy3 in the osteoclast cells is shown in Figure 5B. The merged image shows NB-siRNA-Cy3 (red) being taken up by a multinucleated osteoclast (blue). The NB-250 nM siRNA-Cy3 was distributed around the nuclei, demonstrating intracellular NB-siRNA trafficking.

**Figure 6:**
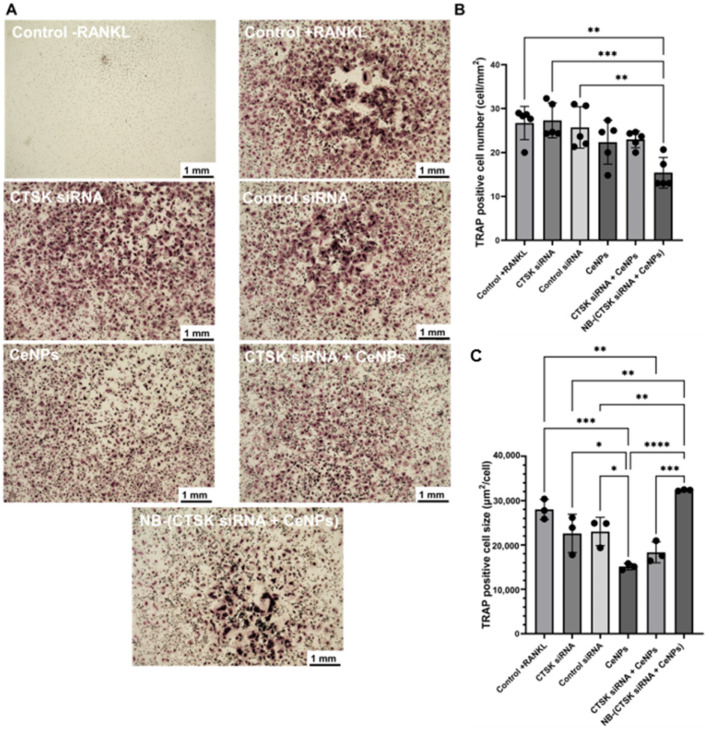
(**A**) TRAP staining of human osteoclasts precursors cultured with the following samples at day 7: control negative (control −RANKL), control positive (control +RANKL), 2.5 nM CTSK siRNA, 2.5 nM control siRNA, 1 µg/mL CeNPs, 1 µg/mL CeNPs + 2.5 nM CTSK siRNA, NB-(2.5 nM CTSK siRNA + 1 µg/mL CeNPs). All of the groups that contained CTSK siRNA and control siRNA were treated with a 1 vol.% transfection reagent. Quantification of TRAP-positive osteoclasts: (**B**) TRAP-positive cell number and (**C**) TRAP-positive cell size. Significant differences were determined using a one-way ANOVA and Tukey’s post hoc test. *: *p* < 0.05, **: *p* < 0.01, ***: *p* < 0.001, ****: *p* < 0.0001.

TRAP staining was performed after 7 days, and the images (Figure 6A) and quantitative results comparing TRAP-positive cell number (cell/mm^2^) and the TRAP-positive cell size (µm^2^/cell) are presented in Figure 6B,C. Except for the −RANKL group, which was not treated with RANKL as a negative control (Figure 6A), osteoclasts were formed in all of the groups (dark red stains in Figure 6A). As shown in Figure 6B, a lower osteoclast TRAP-positive cell number was observed for NB-(CTSK siRNA + CeNPs) (15.4 ± 3.49 cell/mm^2^) compared to the +RANKL control group (26.73 ± 3.80 cell/mm^2^) (*p* < 0.01). However, there was no significant difference among the CTSK siRNA (27.33 ± 3.99 cell/mm^2^), control siRNA (25.73 ± 4.74 cell/mm^2^), CeNPs (22.36 ± 4.99 cell/mm^2^), CTSK siRNA + CeNPs (23 ± 1.90 cell/mm^2^), and the +RANKL control group (*p* > 0.05). TRAP-positive cell size was smaller for the CeNPs (15.1 ± 0.7 × 10^3^ µm^2^/cell) and CTSK siRNA + CeNPs (18.3 ± 2.4 × 10^3^ µm^2^/cell) compared to the +RANKL control group (28 ± 2.3 × 10^3^ µm^2^/cell) (*p* < 0.001), while there was no significant difference among the CTSK siRNA (22.6 ± 4.3 × 10^3^ µm^2^/cell), control siRNA (23 ± 3.2 × 10^3^ µm^2^/cell), and the +RANKL control group (*p* > 0.05). On the other hand, the TRAP-positive cell size of NB-(CTSK siRNA + CeNPs) increased to 32.3 ± 0.2 × 10^3^ µm^2^/cell, which is not significant vs. +RANKL (*p* > 0.05).

## 4. Discussion

The aim of this study was to investigate the effect of loading and releasing a mixture of CTSK siRNA and CeNPs from NB in vitro to evaluate their potential for use in osteoporosis treatment strategies. For loading, a sonication probe was used with a low frequency of 20 kHz, and the siRNA was emulsified in PFC and stabilized with albumin to form the nanobubbles [31]. However, for the release, the nanobubbles were stimulated with a low-intensity pulsed ultrasound (LIPUS). The synthesized nanobubbles had a PFC core that vaporized in response to LIPUS stimulation and subsequently expanded and ruptured, allowing the loaded siRNA to be released [31]. The fluorescence microscope and TEM images showed that the CeNPs were adsorbed to the surface of NB rather than being encapsulated (Figure 2 and Figure 3A). Moreover, according to our previous study [31], the biodegradability of the nanobubbles without LIPUS stimulation was 21.2% after 15 days at 37 °C and 7.4 pH. In this study, the nanobubbles served as a carrier to deliver a mixture of Cathepsin K (CTSK) siRNA and cerium oxide nanoparticles (CeNPs) to inhibit CTSK secretion by osteoclast cells, which triggers osteoporosis and enhances mineralization with antioxidant properties of CeNPs.

The particle size, chemical state, and superoxide dismutase activity (SOD) of the CeNPs and the mixture of CTSK siRNA/CeNPs were similar (the SOD activity was 15 ± 2% for 5 µg/mL CeNPs and 16 ± 2% for CTSK siRNA mixed with 5 µg/mL CeNPs). So, the addition of CTSK siRNA did not affect the antioxidant ability of the CeNPs. Mesenchymal stem cells (MSCs) have remarkable regenerative potential to repair various tissue injuries [62]. Our studies involved treating hMSCs with different NB concentrations (0.25, 0.5, 1, and 5 mg/mL) and demonstrated no toxic effects. Compared to the other NB formulations, which showed high toxicity with the low lipid concentration (5 µg/mL) [63], our NB demonstrated cytotoxicity at 1 mg/mL. However, in another study, a NB made of soy lipid and Tween 80 was not toxic to the human breast cancer cell line at 40 mg/mL [64]. The results of our study showed that the mixture of CTSK siRNA and CeNPs released from NB improved osteogenic differentiation due to the increased mineral deposition (ARS staining; Figure 5A) in the hMSCs. Our ARS results indicated increased mineralization for CTSK siRNA + CeNPs and NB-(CTSK siRNA + CeNPs), which could be attributed to the synergistic action of CeNPs and CTSK siRNA because CeNPs and CTSK siRNA individually did not increase the mineralization compared with the control hMSCs. In another study, CeNPs that had been incubated for 19 days with mouse bone marrow stromal cells revealed a decrease in mineralized nodules [65]. Comparing the results of NB-(CTSK siRNA + CeNPs) and CTSK siRNA + CeNPs showed that NB acted as an applicable delivery system to inhibit osteoclast differentiation (TRAP staining, Figure 6). Notably, only NB-(CTSK siRNA + CeNPs) showed significantly fewer TRAP-positive cells compared to the +RANKL control, which could be attributed to the gradual release and internalization of CTSK siRNA and CeNPs from NB compared to the CTSK siRNA + CeNPs group. Although the TRAP-positive cell size of NB-(CTSK siRNA + CeNPs) is insignificant vs. +RANKL, the lower cell number indicates the suppression of osteoclast formation. Selinger et al. [66] demonstrated that, despite having a similar shape to TRAP-positive cells following CTSK siRNA transfection, the osteoclasts in their study were active, and the bone resorption pits were 60% lower on dentine. Thus, although the osteoclast number of the CTSK siRNA and CTSK siRNA + CeNPs groups were not significantly lower than the +RANKL control group, it is probable that these osteoclasts were inactive; thus, further analyses are required to measure the bone resorption activity of osteoclasts. Comparing the results of our ARS and TRAP staining, CTSK siRNA and CeNPs released from NB are more effective than each component separately or their mixture in the mineralization of hMSCs and downregulation of osteoclastogenesis.

Protecting siRNA plays a key role in effective delivery. For instance, in another study, mesoporous silica nanoparticles were used to protect and deliver siRNA to silence the SOST gene via bone marrow injection in mice models to inhibit sclerostin, which inhibits a signaling pathway for bone remodeling [13]. Also, in a different study, gold nanoparticles decorated with CTSK siRNA were coated on a titanium bone implant, and the release of CTSK siRNA enhanced osteogenesis and reduced osteoclast differentiation due to CTSK deletion in osteoclast [15]. An example of systemic delivery can be seen in the tail vein injection of Plekhol siRNA in the ovariectomized (OVX) rats in [67], which targeted osteoblasts and improved bone regeneration. The bone mineral density of healthy and OVX rats increased after a weekly injection of 3.75 mg/kg siRNA for 9 weeks. However, CTSK was found to be released from other soft tissues, and untargeted delivery can cause cerebrovascular accidents [68]. Hence, targeting CTSK siRNA to the bone is desirable and, this was studied by the authors of [69], who designed an Adeno-associated virus, which is a complex process.

Recent research suggests that several additional factors can influence osteoporotic development. These factors include sustained oxidative stress [70], the activation of macrophages [71], and the prolonged release of pro-inflammatory mediators [72]. Moreover, previous studies involving CeNPs have shown promising results, indicating that CeNPs can mimic cellular antioxidants, scavenge reactive oxygen species (ROS), and exert therapeutic effects in vitro and in vivo across various tissue types [73,74]. The prominent characteristic of CeNPs is their ability to exist in multiple valence states, including the Ce^4+^-oxidized state and the Ce^3+^-reduced state. This feature endows CeNPs with catalase- and superoxide dismutase (SOD)-mimetic activity. You et al. reported that CeNPs in a higher Ce^4+^-oxidized state demonstrated increased osteogenic differentiation compared to CeNPs in a higher Ce^3+^ concentration [75]. However, a recent study by Wei et al. also showed that CeNPs with higher Ce^3+^ surface sites exhibited superior effects in modulating the fate of hMSCs and promoting bone deposition in vitro. This suggests that both the Ce^4+^ and Ce^3+^ states of CeNPs can play crucial roles in influencing cellular responses and bone formation. In addition, the authors of [76] showed that adding nanoceria to bioactive glass foam provoked osteoblast differentiation and collagen forming without osteogenic media. Moreover, in another study, ceria microparticles enhanced the catalysis effect of bioactive glass powder and reduced oxidative stress [77]. Coated Ti implants with nanoceria showed higher osteointegration by forming new bone around the implants in a rat model. New bone formation was attributed to both increased osteogenic properties and the anti-inflammatory effect of nanoceria in [78]. CeNPs introduced to mesoporous silica improved the osteogenic properties of preosteoclast cells, increased mineralization, and inhibited osteoclast activity by scavenging free oxygen radicals in [45]. In addition to the osteogenic ability of CeNPs, they can be used for treating rheumatoid arthritis when anchored with manganese ferrite, which reduces ROS and generates oxygen to polarize inflammatory macrophages to anti-inflammatory ones [79]. Recently, it was shown that CeNPs not only increase osteogenic differentiation but also have a healing effect against ionizing irradiation (IR) [80].

The addition of CTSK siRNA to CeNPs did not significantly affect CeNPs’ physical or chemical properties. The size and morphological character of the CeNPs and mixture of CTSK siRNA/CeNPs, which were determined via TEM analysis, are in line with the observations reported in our previous works regarding CeNPs produced via a similar synthesis [1,3,4]. The deconvolution of the fitted peaks in XPS related to Ce^3+^ and Ce^4+^ states shows that the mixture of the CTSK siRNA and CeNP sample components does not significantly change the surface cerium redox state distribution of CeNPs. The similar SOD activities of the CeNPs and the CTSK siRNA/CeNPs mixture suggests that the reactive oxygen species (ROS) scavenging activity noted for CeNPs was largely retained after CTSK siRNA addition. Hydroxyl radicals (HO•), superoxide anions (O_2_^−^), and hydrogen peroxide are common ROS molecules produced in the body under various conditions. The pathological dysregulation of ROS can cause bone damage and cell death [5,6,7,8,9,10]. CeNPs engineered to possess a high fraction of reduced surface sites have been shown to ameliorate these oxidative stresses and promote cell survival and proliferation. Therefore, we have considered using a high Ce^3+^-fraction CeNPs formulation to promote bone cell formation. Previous studies have demonstrated a tendency towards bone formation upon treatment with a given nanoparticle formation.

In the present study, we considered the effects of the CTSK siRNA/CeNPs mixture via a combinatorial approach. This mixture can be administered intravenously using an ultrasound-responsive nanobubble platform. However, oral administration would be beneficial, and the effect of pH on the release rate should be investigated. CeNPs elicited an osteogenic effect, promoting cell health and mineralization, while CTSK siRNA reduced osteoclast formation through the local delivery method (ultrasound). However, for a translational approach, it is necessary to study the efficiency of this method in vivo in a ovariectomized (OVX) mice model to evaluate the potential and time response for osteoporosis treatment. Moreover, more biomolecular tests are necessary to investigate the gene-silencing effect and mechanisms. Also, it is necessary to increase the AE% by modifying the adsorption method and monitoring the release test for a more extended period, and this is something which we may consider for our next study. The measurement of the zeta potential of the nanobubbles will be investigated in future to gain a better understanding of the system properties. Due to the systemic nature of osteoporosis, treating it requires specialized equipment with multiple ultrasound stimulation probes capable of penetrating the entire skeletal system without harming the surrounding tissues.

## 5. Conclusions

A mixture of CTSK siRNA and CeNPs was loaded onto a ultrasound-responsive nanobubble. The mixture of siRNA and CeNPs did not significantly affect the physical and chemical properties of the CeNPs, including the oxidative stress activity. The adsorption efficiency (AE%) for NB-CTSK siRNA was 25.44 ± 5.38%, and the adsorption efficiency for the NB-CeNPs was 17.28 ± 1.94%. The release of 2.32 ± 0.55 nM for CTSK siRNA and 0.97 ± 0.09 µg/mL for CeNPs from 1 mg/mL of NB was detected after 3 days post-ultrasonication. The mineralization of hMSCs was increased by more than two-fold for NB-(CTSK siRNA + CeNPs) (1.12 ± 0.03) compared to the control hMSCs (0.41 ± 0.02). Although the TRAP-positive cell number of NB-(CTSK siRNA + CeNPs) (15.4 ± 3.49 cell/mm^2^) decreased compared to the +RANKL control group (26.73 ± 3.80 cell/mm^2^) (*p* < 0.01), there was no significant difference between the cell size values of these two groups. Overall, a US-responsive nanobubble system could facilitate a promising method for the combined delivery of CTSK siRNA and CeNPs to treat bone cells.

## Data Availability

All relevant data are contained in the present manuscript.
